# HDAC6 Regulates the MRTF-A/SRF Axis and Vascular Smooth Muscle Cell Plasticity

**DOI:** 10.1016/j.jacbts.2018.08.010

**Published:** 2018-12-31

**Authors:** Mengxue Zhang, Go Urabe, Christopher Little, Bowen Wang, Alycia M. Kent, Yitao Huang, K. Craig Kent, Lian-Wang Guo

**Affiliations:** a Department of Surgery and Department of Physiology and Cell Biology, College of Medicine, and the Davis Heart and Lung Research Institute, Wexner Medical Center, The Ohio State University, Columbus, Ohio; b Cellular and Molecular Pathology Graduate Program, School of Medicine and Public Health, University of Wisconsin, Madison, Wisconsin; c Department of Surgery, College of Medicine, and the Davis Heart and Lung Research Institute, Wexner Medical Center, The Ohio State University, Columbus, Ohio; d Department of Surgery, School of Medicine and Public Health, University of Wisconsin, Madison, Wisconsin

**Keywords:** dedifferentiation, HDAC6, MRTF-A, SRF, vascular smooth muscle cell, SMA, smooth muscle actin, DMEM, Dulbecco’s modified Eagle’s medium, DNA, deoxyribonucleic acid, EEL, external elastic lamina, FBS, fetal bovine serum, HDAC, histone deacetylase, IEL, internal elastic lamina, IgG, immunoglobulin G, IH, intimal hyperplasia, MMP, matrix metalloproteinase, MRTF-A, myocardin-related transcription factor A, PDGF-BB, platelet-derived growth factor-BB, siRNA, small interfering ribonucleic acid, SMC, vascular smooth muscle cell, SMHC, smooth muscle myosin heavy chain, SRF, serum response factor, TNF, tumor necrosis factor, TSA, trichostatin A

## Abstract

Cellular plasticity is fundamental in biology and disease. Vascular smooth muscle cell (SMC) dedifferentiation (loss of contractile proteins) initiates and perpetrates vascular pathologies such as restenosis. Contractile gene expression is governed by the master transcription factor, serum response factor (SRF). Unlike other histone deacetylases, histone deacetylase 6 (HDAC6) primarily resides in the cytosol. Whether HDAC6 regulates SRF nuclear activity was previously unknown in any cell type. This study found that selective inhibition of HDAC6 with tubastatin A preserved the contractile protein (alpha-smooth muscle actin) that was otherwise diminished by platelet-derived growth factor-BB. Tubastatin A also enhanced SRF transcriptional (luciferase) activity, and this effect was confirmed by HDAC6 knockdown. Interestingly, HDAC6 inhibition increased acetylation and total protein of myocardin-related transcription factor A (MRTF-A), a transcription co-activator known to translocate from the cytosol to the nucleus, thereby activating SRF. Consistently, HDAC6 co-immunoprecipitated with MRTF-A. In vivo studies showed that tubastatin A treatment of injured rat carotid arteries mitigated neointimal lesion, which is known to be formed largely by dedifferentiated SMCs. This report is the first to show HDAC6 regulation of the MRTF-A/SRF axis and SMC plasticity, thus opening a new perspective for interventions of vascular pathologies.

## Highlights

• Distinct from other histone deacetylases, HDAC6 primarily resides in the cytosol.
• Unexpectedly, HDAC6-selective inhibition (or silencing) enhances the nuclear activity of SRF.
• HDAC6 inhibition elevates acetylation and protein levels of myocardin-related transcription factor A, a cytoplasmic-nuclear shuttling co-activator of SRF. Myocardin-related transcription factor A/SRF are known to critically regulate vascular smooth muscle cell phenotypic stability.
• HDAC6 inhibition prevents smooth muscle cell dedifferentiation in vitro and reduces neointima and restenosis in vivo.

## Visual Abstract

**Figure:**
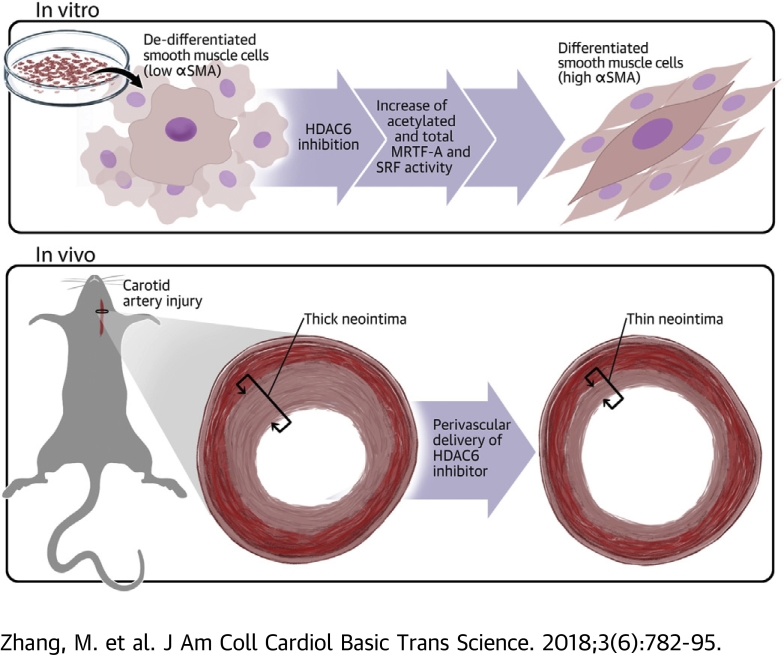

It is well documented that even highly differentiated mature vascular smooth muscle cells (SMCs) remain plastic (1). Upon mechanical injury or exposure to chemical/biological stimuli, SMCs undergo phenotypic transformation. Among acquired phenotypes, a hallmark of transformed SMCs is the loss of SMC signature; that is, the decline of contractile proteins, including α-smooth muscle actin (SMA) and smooth muscle myosin heavy chain (SMHC), and consequently compromised contractile function (2). This SMC phenotypic transformation is central to the buildup of neointimal lesions, which directly cause flow obstruction in cardiovascular diseases such as atherosclerosis and restenosis.

SMC transformation is perpetrated by altered expression of an array of genes. Epigenetic remodeling is increasingly recognized as playing a crucial role in this process (2). Histone deacetylases (HDACs) remove epigenetic marking (histone lysine acetylation), thereby critically regulating transcription. Four classes of HDACs have been identified thus far: class I (HDACs 1, 2, 3, and 8), class IIa (HDACs 4, 5, 7, and 9) and class IIb (HDACs 6 and 10), class III (sirtuins 1 through 7), and class IV (HDAC 11). Aberrant HDAC activities are implicated in a wide range of disease conditions. Some HDAC inhibitors are in clinical trials; a few have been approved by the U.S. Food and Drug Administration (3). HDAC6 is unusual because of its unique domain composition and primary cytosolic localization in most cells, in which its enzymatic activity modulates cytoskeleton and other proteins in the cytosol (4). Unlike other HDACs, HDAC6 regulation of transcription factor nuclear activity has rarely been reported 5, 6, 7.

HDACs were not investigated in vascular cells until relatively recently 2, 8, 9, 10. Most of the studies are pharmacology-based using pan inhibitors that do not distinguish individual HDACs. Likely for this reason, reports are often contradictory, even using the same HDAC inhibitor in the same animal or cellular model. For example, trichostatin A (TSA), a pan inhibitor of class I and class II HDACs, was reported to abate neointima in the balloon injury model of rat carotid artery (11). However, in another report using the same model, TSA potentiated neointima formation (12). Opposite effects of TSA were also reported on proliferation/migration of primary rat SMCs 12, 13, 14. Moreover, whereas earlier reports indicated that HDACs 2, 4, and 5 repress SMC contractile gene expression (15), a more recent study showed that TSA treatment diminished SMC contractile proteins in human primary cells (16).

These disparities inspired us to explore differential functions of individual HDACs. We chose the unique HDAC6 from class IIb and HDAC3 from class I for contrast. Whereas HDAC3 is typically chromatin-associated, HDAC6 is essentially a cytosolic protein although its nuclear presence has also been reported (7). Both are implicated in vascular diseases; however, the knowledge of their regulations of SMC marker (contractile) proteins is conspicuously lacking. Our data show that HDAC6 inhibition elevates, whereas HDAC3 inhibition reduces, SMC marker protein levels. With an initial objective to compare these 2 HDACs in SMC phenotypic transformation, we made a surprising finding that inhibition of HDAC6, known as a cytosolic enzyme, robustly increased the nuclear activity of serum response factor (SRF). This result could be rationalized by another novel finding reported here; that is, HDAC6 inhibition elevated acetylation and total protein of myocardin-related transcription factor A (MRTF-A), a master regulator that can activate SRF-directed contractile gene transcription if undergoing a cytosol–nucleus translocation. Our findings are unexpected also because there are few reports on HDAC6 modulation of transcription factor nuclear activities 6, 7. In addition, our in vivo data show that a selective HDAC6 inhibitor (but not HDAC3 inhibitor) effectively reduced neointima in a rat vascular injury model. As such, the present study highlights the unique HDAC6 regulatory mechanism in SMC pathobiology that could be targeted in vivo for neointima mitigation.

## Methods

### Cell and artery explant cultures and pretreatment with HDAC inhibitors

The mouse aortic smooth muscle MOVAS cell line was purchased from ATCC (Manassas, Virginia), and the cell culture was maintained at 37°C and 5% CO_2_ in Dulbecco’s modified Eagle’s medium (DMEM) supplemented with 10% fetal bovine serum (FBS) (Thermo Fisher Scientific, Waltham, Massachusetts). Cells at passage 25 were used for experiments. For pretreatment with HDAC inhibitors, MOVAS cells were starved overnight with 0.5% FBS and then added with an HDAC inhibitor 2 h before stimulation with platelet-derived growth factor-BB (PDGF-BB) (20 ng/ml) or tumor necrosis factor (TNF)-α (20 ng/ml), both human recombinant (R&D Systems, Minneapolis, Minnesota). For ex vivo treatment, rat (see later discussion) aortas deprived of endothelium were cut into strings and cultured in DMEM/F12 containing 0.5% FBS. After a 24-h incubation in the presence of vehicle or 10 μmol/l tubastatin A, the artery explants were pooled and homogenized for immunoprecipitation and Western blotting.

### Assays for cell proliferation and migration

Proliferation was determined by using the CellTiter-Glo Luminescent Cell Viability kit (Promega, Madison, Wisconsin) following manufacturer’s instructions, as previously described (17). MOVAS cells were seeded in 96-well plates at a density of 2,000 cells per well with a final volume of 200 μl DMEM (10% FBS) and grown for 6 h. Cells were then starved with 0.5% FBS overnight and pretreated for 2 h with a series of concentrations of tubastatin A (ApexBio, Boston, Massachusetts), RGFP966 (ApexBio), or vehicle control (equal volume of dimethylsulfoxide) mixed in fresh starvation medium before PDGF-BB mitogenic stimulation. At 72 h of PDGF-BB treatment, plates were decanted, refilled with 50 μl CellTiter-Glo reagent/50 μl phosphate-buffered saline per well, and incubated at room temperature for 10 min before reading in a FlexStation 3 Benchtop Multi-Mode Microplate Reader (Molecular Devices, San Jose, California) (250-ms integration).

To determine cell migration, scratch (wound healing) assay was performed as described in our previous report (17). Briefly, SMCs were cultured to a 90% confluency in 6-well plates and then starved overnight with 5 μmol/l tubastatin A, 5 μmol/l RGFP966, or vehicle included. A sterile pipette tip was used to generate an ∼1 mm cell-free gap. Dislodged cells were washed away with phosphate-buffered saline. Plates were then refilled with a medium containing 20 ng/ml of PDGF-BB and 5 μmol/l of tubastatin A (or RGFP966) and incubated for 24 h. Calcein AM was then added (final 2 μmol/l) to illuminate the cells. After 15-min incubation, cells were washed 3 times with phosphate-buffered saline, and images were taken. Cell migration was quantified by using ImageJ software (National Institutes of Health, Bethesda, Maryland) based on the change in the width of the cell-free gap before and after PDGF-BB stimulation.

### Quantitative real-time polymerase chain reaction to determine expression levels of inflammatory cytokines

Assays were performed following our published methods (17). Briefly, total ribonucleic acid was isolated from cultured cells by using a Trizol reagent (Thermo Fisher Scientific) following the manufacturer's protocol. Potential contaminating genomic deoxyribonucleic acid (DNA) was removed by using gDNA Eliminator columns provided in the kit. Total ribonucleic acid of 1 μg was used for the first-strand complementary DNA synthesis (Thermo Fisher Scientific). Quantitative real-time polymerase chain reaction was performed by using Quant Studio 3 (Thermo Fisher Scientific). Each complementary DNA template was amplified in triplicate PerfeCTa SYBR Green SuperMix (Quantabio, Beverly, Massachusetts) with gene-specific primers:

MCP-1, Forward: GCTCAGCCAGATGCAGTTAA; Reverse: TCTTGAGCTTGGTGACAAAAACT
MMP2, Forward: CCATCGAGACCATGCGGAAG; Reverse: CCTGTATGTGATCTGGTTCTTG
MMP3, Forward: CAGACTTGTCCCGTTTCCAT; Reverse: GGTGCTGACTGCATCAAAGA
MMP9, Forward: TCATCCAGTTTGGTGTCGCG; Reverse: GACCACAACTCGTCGTCGTC
IL-6, Forward: CCTCTGGTCTTCTGGAGTACC; Reverse: ACTCCTTCTGTGACTCCAGC
GAPDH, Forward: GAGAGTGTTTCCTCGTCCCG; Reverse: ATGGGCTTCCCGTTGATGAC.

### Exogenous HDAC6 expression and endogenous HDAC6 knockdown

The plasmid for expression of FLAG-tagged HDAC6 was purchased from Addgene (Cambridge, Massachusetts; catalog no. 30482), and transfected into HEK293 cells by using JetPRIME (Polyplus-transfection, catalog no. 114-15). For HDAC6 knockdown in SMCs, 3 small interfering ribonucleic acids (siRNAs) of different sequences were ordered from Thermo Fisher Scientific and tested for efficiency. The sequence of the most efficient HDAC6 siRNA is as follows: sense, CCUAGUGUGAUUAUACGUGUTT; antisense, ACACGUAUAAUACACUAGGGT. The siRNA was transfected into MOVAS cells by using RNAiMax Reagent (Thermo Fisher Scientific; catalog no. 13778-075) following the manufacturer’s protocol.

### Luciferase assay for SRF transcriptional activity

We followed the manufacturer’s protocol. Briefly, MOVAS cells were transfected with pGL4.34 Vector plasmids (Promega; catalog no. E1350) using Effectene Transfection Reagent (Qiagen, Germantown, Maryland; catalog no. 301425). Positive cells were selected out by using Hygromycin B (Thermo Fisher Scientific, catalog no. 10687010), seeded in 24-well plates at a density of 20,000 cells/well, and grown for 6 h in DMEM containing high glucose and 10% FBS. Cells were then starved overnight and treated with vehicle or 5 μmol/l tubastatin A for 24 h before lysis in Bright-Glo (Promega; catalog no. 2610) followed by luminescence reading.

### Western blotting to determine changes of protein or acetylation levels

At the end of each treatment, cells were collected and lysed in radio-immunoprecipitation assay buffer containing protease inhibitors (50 mmol/l Tris, 150 mmol/l NaCl, 1% Nonidet P-40, 0.1% sodium dodecyl sulfate, and 10 μg/ml aprotinin). Approximately 15 to 30 μg of proteins from each sample (quantified with a Bio-Rad DC Protein Assay kit, Bio-Rad, Hercules, California) were separated via sodium dodecyl sulfate-polyacrylamide gel electrophoresis on a 10% gel. The proteins were then transferred to a polyvinylidene difluoride membrane and detected by immunoblotting using respective antibodies. The antibody sources and dilution ratios are as follows: rabbit monoclonal anti-α-SMA, Abcam (Cambridge, Massachusetts; catalog no. ab32575), 1:1,000; rabbit polyclonal anti-SMHC, Abcam (catalog no. ab53219), 1:1,000; mouse monoclonal anti–β-actin, Abcam (catalog no. ab6276), 1:1,000; rabbit polyclonal anti–MRTF-A, Cell Signaling Technology (Danvers, Massachusetts; catalog no. 14760S), 1:1,000; rabbit polyclonal anti–Ac-Lysine, Cell Signaling Technology (catalog no. 9441S), 1:1,000; and rabbit polyclonal anti–Ac-αtubulin, Cell Signaling Technology (catalog no. 5335S), 1:1,000. Secondary antibodies were as follows: Goat Anti-Mouse IgG (H + L)-HRP Conjugate (Bio-Rad, Hercules, California, catalog no. 1706516) and Goat Anti-Rabbit IgG (H + L)-HRP Conjugate (Bio-Rad, catalog no. 1706515). Specific protein bands on the blots were illuminated by applying enhanced chemiluminescence reagents (Thermo Fisher Scientific; Catalog no. 32106) and then recorded with an Azur LAS-4000 Mini Imager (GE Healthcare Bio-Sciences, Piscataway, New Jersey). Band intensity was quantified by using ImageJ software.

### Co-immunoprecipitation

At the end of each treatment, cells were collected by using an immunoprecipitation lysis buffer (Thermo Fisher Scientific; catalog no. 87787) containing a protease inhibitor cocktail (Thermo Fisher Scientific; catalog no. 78430), and kept on ice for 30 min to ensure complete lysis. Cell lysates of equal protein amounts were incubated overnight at 4°C (constant rotation) with 1 to 2 μg of an MRTF-A antibody (Cell Signaling Technology; catalog no. 14760S) or immunoglobulin G (IgG) control (Santa Cruz Biotechnology, Dallas, Texas; catalog no. sc2027). Co-immunoprecipitation of MRTF-A with associated proteins was then performed by using Protein A/G Agarose beads included in the Pierce Classic IP Kit (Thermo Fisher Scientific; catalog no. 26146) followed by Western blotting to detect immunoprecipitated MRTF-A or co-immunoprecipitated HDAC6.

### Animals

All animal studies conform to the *Guide for the Care and Use of Laboratory Animals* (National Institutes of Health) and protocols approved by the Institutional Animal Care and Use Committee at The Ohio State University (Columbus, Ohio). Male Sprague-Dawley rats (Charles River Laboratories, Wilmington, Massachusetts) were used.

### Restenosis model of rat carotid artery balloon angioplasty

Male Sprague-Dawley rats (300 to 350 g) underwent balloon injury of the left common carotid artery, as previously described (18). Briefly, after induction of anesthesia with isoflurane, a 2-F balloon catheter (Edwards Lifesciences Corp., Irvine, California) was inserted through the left external carotid artery into the common carotid artery and insufflated with 2 atm of pressure 3 times. The external carotid artery was then ligated, and blood flow was resumed. Rats were euthanized at 14 days after injury. Arteries were collected and processed for sectioning and morphometric characterization.

### Morphometric analysis of neointima

Paraffin sections (5 μm thick) were excised at equally spaced intervals and then stained (van Gieson or hematoxylin and eosin) for morphometric analysis, as described in our previous reports (18). Planimetric parameters as follows were measured on the sections and calculated by using ImageJ software: area inside external elastic lamina (EEL area), area inside internal elastic lamina (IEL area), lumen area, intima area (= IEL area − lumen area), and media area (= EEL area – IEL area). Intimal hyperplasia (IH) was quantified as a ratio of intima area versus media area. Measurements were performed by a student blinded to the experimental conditions using 3 to 6 sections from each of 3 rats in a vehicle control or HDAC inhibitor treatment group. The data from all sections were pooled to generate the mean for each animal. The means from all the animals in each treatment group were then averaged, and the SEM was calculated.

### Immunohistochemistry for detection of α-SMA in the arterial wall

Immunostaining was performed on cross sections following our published method (17). Briefly, the sections were first incubated with the primary rabbit monoclonal anti-α-SMA (Abcam; catalog no. ab32575) for 12 h and rinsed at least 3 times. Normal IgG was used for background control. The α-SMA protein was then visualized by fluorescence microscopy after incubating the sections with an anti-rabbit secondary antibody conjugated with Alexa Fluor 488 (Thermo Fisher Scientific). For quantification, 5 immunostained sections from each animal were used. Fluorescence intensity in each image field was quantified by using ImageJ software and normalized according to the number of 4′,6′-diamidino-2-phenylindole–stained nuclei in the media and neointima. The values from all 5 sections were pooled to generate the mean for each animal. The means from all the animals in each treatment group were then averaged, and the SEM was calculated.

### Statistical analysis

Independent experiments (at least 3 times) were performed to confirm the same result. Data are presented as mean ± SEM. As specifically stated in each figure legend, Student’s *t*-tests or 1-way analysis of variance followed by Tukey's honest significant difference post hoc tests were performed; p values <0.05 were considered statistically significant.

## Results

### Whereas HDAC6 inhibition increases, HDAC3 inhibition reduces the α-SMA protein

**Figure 1:**
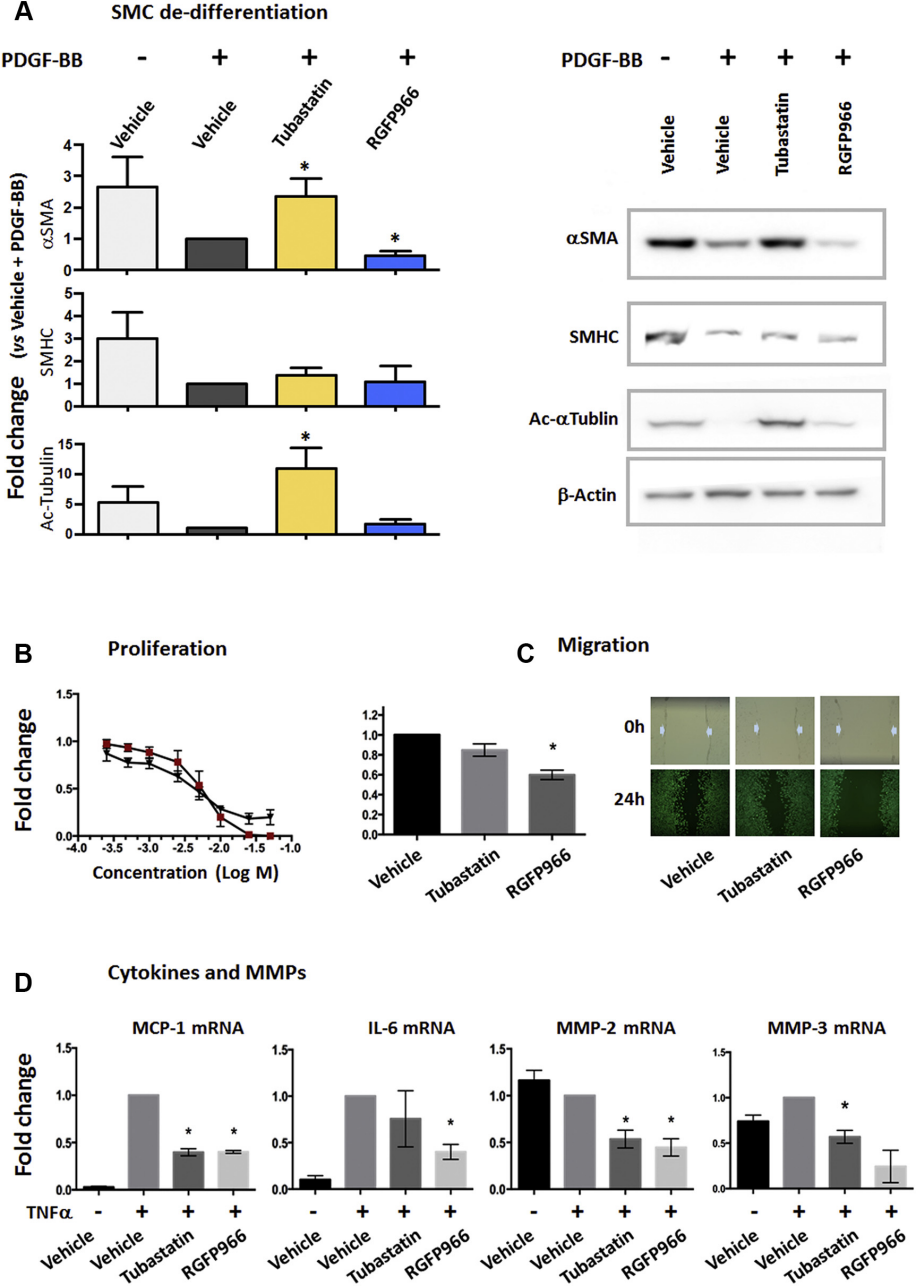
Up-Regulation of SMC Marker Proteins by HDAC6 Inhibition Under PDGF-BB Stimulation MOVAS cells were starved with 0.5% fetal bovine serum for overnight, and then pretreated with 5 μmol/l tubastatin A or 5 μmol/l RGFP966 or vehicle control (equal amounts of dimethylsulfoxide) for 2 h before the addition of 20 ng/ml of platelet-derived growth factor-BB (PDGF-BB). Data are quantified as fold changes versus control (normalized value as 1, condition specified later); mean ± SEM; n = 3 independent experiments; *p < 0.05 compared with control (one-sample Student’s *t*-test). **(A)** Western blot assay of vascular smooth muscle cell (SMC) markers. Cells were collected 48 h after PDGF-BB stimulation. Shown on the **right side** are representative blots from the same polyacrylamide gel. Control: vehicle + PDGF; normalization to β-actin. **(B)** Dose response of proliferation inhibition. CellTiter-Glo assays were performed 72 h after PDGF stimulation. The basal level reading (i.e., 72 h after adding solvent [control to PDGF-BB]) was subtracted. Control: vehicle, 72 h PDGF stimulation. **Red square** = tubastatin; **black triangle** = RGFP966. **(C)** Migration measured with scratch assay. Pictures show the scratch gaps before (0 h) and after (24 h) PDGF-BB stimulation. Control: vehicle + PDGF. **(D)** Quantitative real-time polymerase chain reaction assay. MOVAS cells were pretreated with vehicle or inhibitors for 2 h and then stimulated with tumor necrosis factor α (TNFα) for 4 h. Control: vehicle + TNFα; normalization to glyceraldehyde-3-phosphate dehydrogenase. SMA = smooth muscle actin; HDAC6 **=** histone deacetylase 6; IL-6 interleukin-6; MCP-1 = monocyte chemoattractant protein-1; MMP = matrix metalloproteinase; mRNA = messenger ribonucleic acid; SMHC = smooth muscle myosin heavy chain.

A hallmark of SMC phenotypic transformation is its dedifferentiation, commonly monitored as a decline in SMC contractile proteins. Whether or how HDAC6 (or HDAC3) regulates SMC dedifferentiation has never been clearly addressed. Here we determined the impact of isoform-specific HDAC inhibitors on the SMC contractile proteins α-SMA and SMHC. Tubastatin A is a high-affinity (half maximal inhibitory concentration: 15 nM) HDAC6 inhibitor that is >1,000-fold selective over other HDACs. RGFP966, a novel HDAC3 inhibitor (half maximal inhibitory concentration: 80 nmol/l), exhibits at least 200-fold selectivity over the rest of the HDACs. We applied these selective inhibitors to an established mouse SMC line (MOVAS) that was stimulated with PDGF-BB to induce SMC phenotypic transformation. To our surprise, pretreatments with tubastatin A and RGFP966 produced nearly opposite effects on the expression of SMC markers. As shown in Figure 1A, PDGF-BB treatment substantially down-regulated both α-SMA and SMHC in SMCs, as generally observed in the literature (15). Interestingly, pre-treatment with tubastatin A (5 μ) averted PDGF-induced down-regulation of α-SMA, maintaining it at the basal level (no PDGF-BB). Tubastatin A also increased SMHC, albeit not to a statistically significant level. In contrast, RGFP966 did not increase but further reduced PDGF–down-regulated α-SMA (also see Supplemental Figure 1), although this inhibitor did not change SMHC levels under PDGF-BB treatment. Furthermore, supporting the specificity of tubastatin A for HDAC6 inhibition, tubastatin A (but not RGFP966) dramatically enhanced α-tubulin acetylation (Supplemental Figure 2), which is a well-documented function of HDAC6 4, 7.

### HDAC6 and HDAC3 inhibitors attenuate SMC proliferation, migration, and inflammation

In contrast to differentiated SMCs, aberrantly transformed SMCs exhibit multiple acquired phenotypes, including proliferation, migration, and production of inflammatory cytokines in addition to dedifferentiation. These derivative behaviors critically contribute to vascular pathology, particularly the development of neointima (17). We thus next determined how HDAC6 and HDAC3 influence these pathogenic phenotypes. Our data show that both tubastatin A and RGFP966 inhibited PDGF-stimulated SMC proliferation and migration (Figures 1B and 1C). Although the inhibition of SMC proliferation by tubastatin A was slightly more effective than that by RGFP966, PDGF-stimulated SMC migration was better attenuated by RGFP966 than by tubastatin A of the same concentration (5 μmol/l).

We also measured the expression of pro-inflammatory chemokines and cytokines as well as matrix metalloproteinases (MMPs) because their expression levels often change concomitantly with inflammation (Figure 1D). All these markers constitute SMC inflammatory metrics and have not been previously determined for the effect of either tubastatin A or RGFP966. Although both inhibitors equally inhibited TNFα-induced expression of monocyte chemoattractant protein-1 and MMP2, RGFP966 seemed to be more effective than tubastatin A in suppressing the expression of interleukin-6 and MMP3. Interestingly, both inhibitors further increased TNFα-induced MMP9 expression to similar extents (Supplemental Figure 3). The differential response of MMP9 in contrast to that of MMP2 and MMP3 underscores a gene-specific effect of HDAC6 (or 3) inhibition.

Thus, our data show that tubastatin A and RGFP966 inhibited SMC proliferation, migration, or cytokine/chemokine expression with largely similar patterns. This result is in contrast to the drastically different effects of tubastatin A and RGFP966 on the expression of SMC markers.

### HDAC6 inhibition elevates SRF transcriptional activity in SMCs

As indicated by the aforementioned results, inhibition of HDAC6 (but not HDAC3) mitigates the pathogenic phenotypes of transformed SMCs in a full spectrum: not only proliferation, migration, and inflammation but also dedifferentiation. Because SMC dedifferentiation is a fundamental change that influences other phenotypes, the benefit of HDAC6 inhibition in maintaining the differentiated state (contractile gene expression) of SMCs is of particular interest. We therefore next focused on investigating the underlying molecular mechanisms.

**Figure 2:**
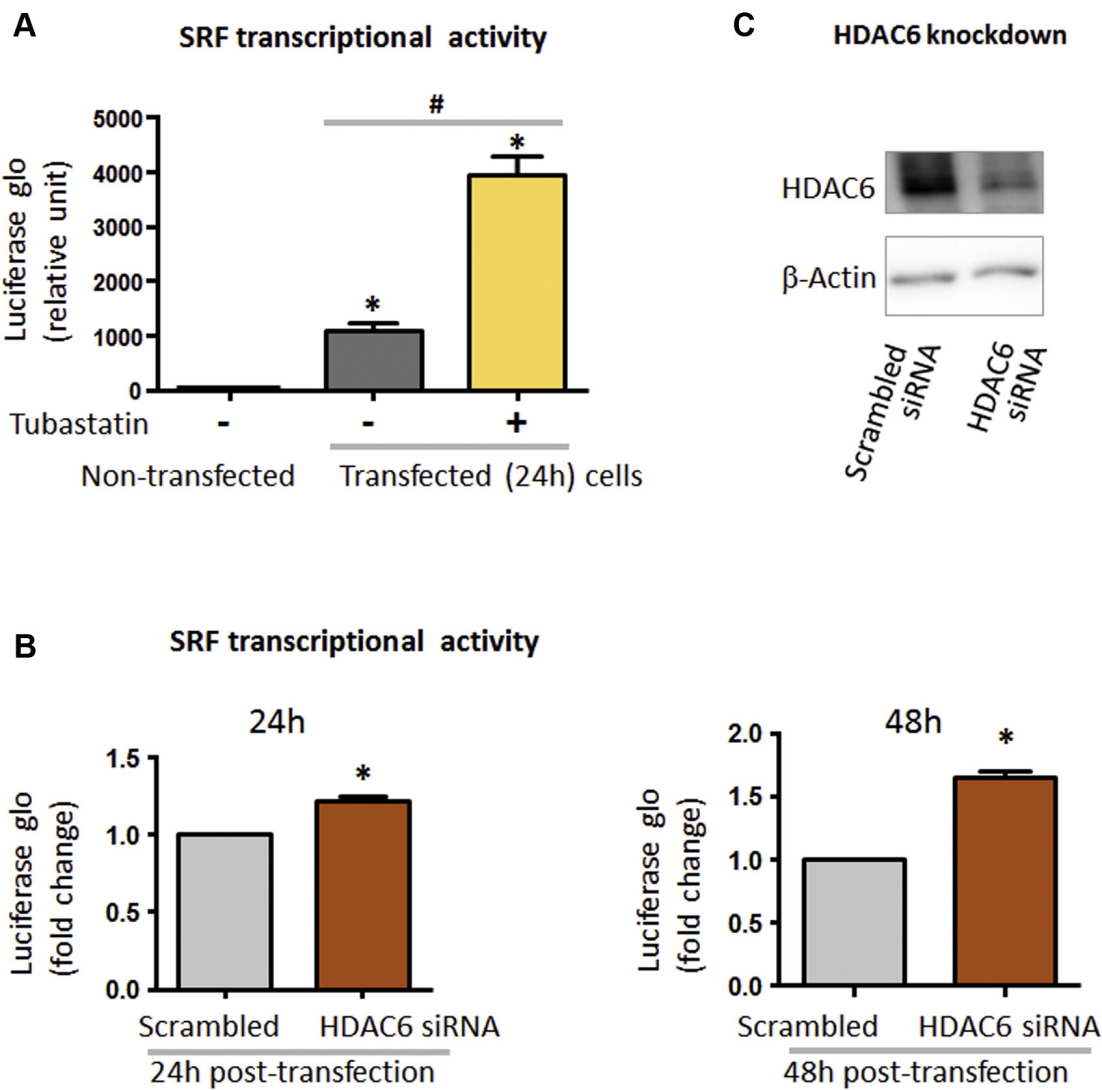
Enhancement of SRF Transcriptional Activity by HDAC6 Inhibitor and siRNA Silencing **(A, B)** Luciferase assay for serum response factor (SRF) transcriptional activity. **(C)** Western blots showing efficient histone deacetylase 6 (HDAC6) knockdown. For luciferase assay, MOVAS cells in 10% fetal bovine serum (FBS) culture were transfected with the E1350 construct for 6 h. Transfected cells were selected with hygromycin B, recovered in Dulbecco’s modified Eagle’s medium (DMEM) containing high glucose and 10% FBS for 24 h, and then seeded in 24-well plates at a density of 20,000 cells/well. After overnight starvation in DMEM (high glucose, 0.5% FBS), cells were **(A)** treated with vehicle or 5 μmol/l tubastatin A for 24 h or **(B)** transfected with scrambled small interfering ribonucleic acid (siRNA) or HDAC6-specific siRNA for 24 h or 48 h, followed by lysis in Bright-Glo for luciferase assay. For each bar graph, at least 3 independent experiments were performed; mean ± SEM; *p < 0.05 compared with **(A)** nontransfected or **(B)** scrambled control, ^#^p < 0.05 compared between vehicle and tubastatin A, analyzed with 1-sample Student’s *t*-test.

SMC contractile gene expression is controlled by the master transcription factor SRF. We thus determined whether HDAC6 inhibition changes SRF transcriptional activity, using the well-established SRF luciferase reporter gene assay (19). As shown in Figure 2A, transfected MOVAS cells exhibited a 1,000-fold increase of luciferase signal compared with the background signal of nontransfected cells, validating the assay method. Interestingly, HDAC6 inhibition with tubastatin A increased luciferase activity by 4-fold compared with control cells treated with vehicle. These data show that selective HDAC6 inhibition elevated SRF transcriptional activity.

Although tubastatin A is >1,000-fold selective for HDAC6 over all other HDACs, the tubastatin A selectivity for HDAC6 over HDAC8 (in class I) is only 57-fold. To further confirm that the observed effect of tubastatin A was truly mediated by HDAC6, siRNA was used to knock down HDAC6 (Figures 2B and 2C). Indeed, HDAC6 silencing largely recapitulated the effect of tubastatin A on the enhancement of SRF transcriptional activity (48 h after siRNA transfection) (Figure 2B).

Taken together, our data show for the first time that either pharmacologically inhibiting HDAC6 or genetically silencing HDAC6 elevates SRF transcriptional activity in SMCs, a result not previously reported in any cell type.

### HDAC6 inhibition increases MRTF-A acetylation and its total protein

**Figure 3:**
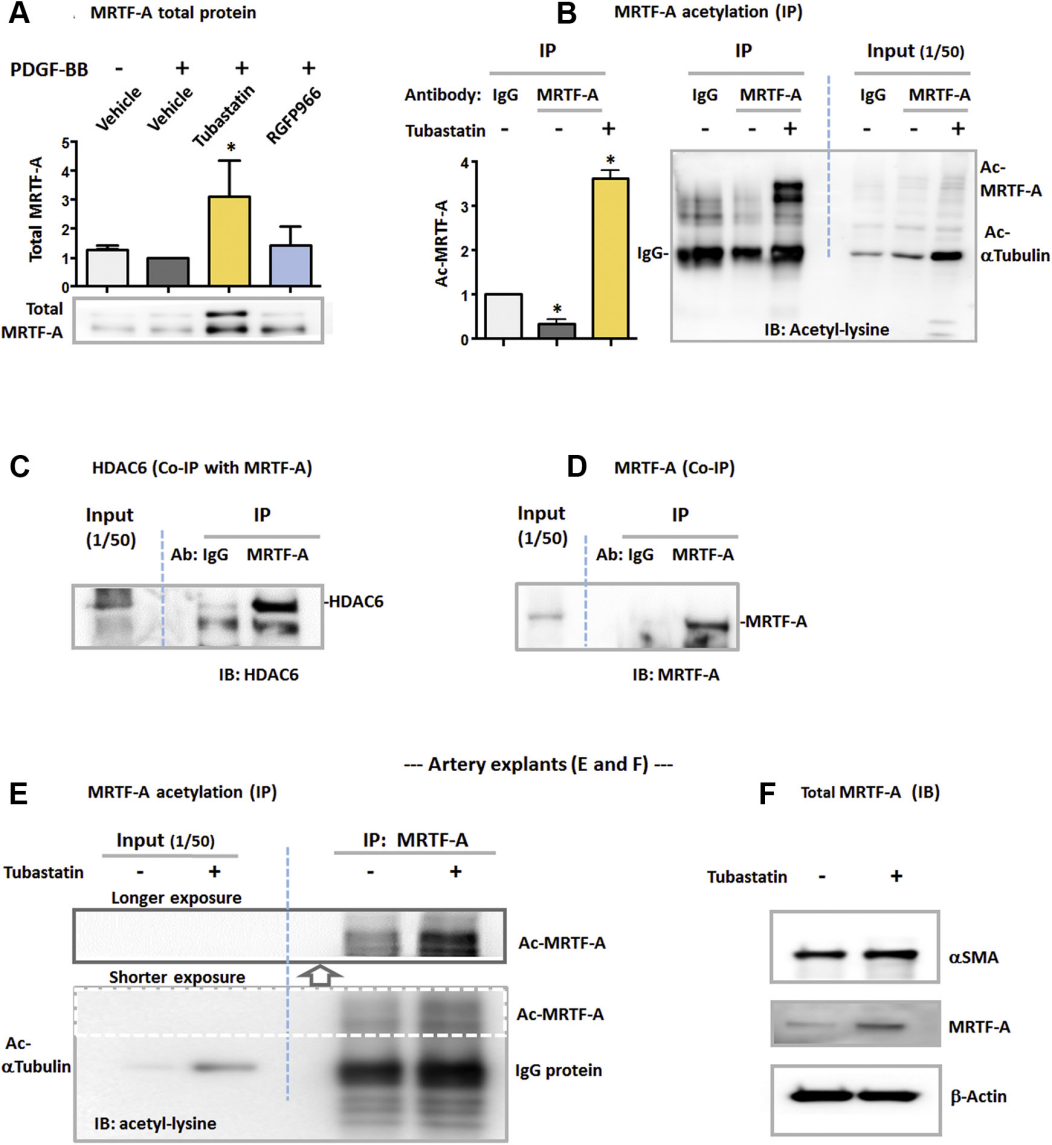
Elevation of MRTF-A Acetylation and Total Protein by HDAC6 Inhibition in Cultured Cells and Rat Artery Explants **(A)** Western blotting of myocardin-related transcription factor A (MRTF-A) total protein. Starved (overnight) MOVAS cells were pretreated with vehicle or HDAC inhibitors for 2 h and then stimulated with PDGF-BB for 48 h, as described in Figure 1A. MRTF-A duplicate bands are generally observed in the literature (25). Data are quantified as fold changes versus control (vehicle + PDGF, normalized value as 1); mean ± SEM; n = 3 independent experiments; *p < 0.05 compared with control (1-sample Student’s *t*-test). **(B)** Western blotting of acetylated MRTF-A. Starved MOVAS cells were incubated with vehicle or 5 μmol/l tubastatin A for 24 h and then collected for immunoprecipitation (IP) by using an MRTF-A antibody or equal amount of immunoglobulin G (IgG) for control. Immunoblotting (IB) was performed to detect acetyl-lysine. **Dashed line** separates IP from Input, both loaded on the same gel. Data are quantified as fold changes versus IgG control (normalized value as 1); mean ± SEM; n = 3 independent experiments; *p < 0.05 compared with control (1-sample Student’s *t*-test). **(C, D)** Co-immunoprecipitation (Co-IP) of HDAC6 with MRTF-A. HDAC6 was overexpressed in HEK293 cells. Equal amounts of IgG and MRTF-A antibody were used for IP. Co-immunoprecipitated proteins were detected by IB for **(C)** HDAC6 or for **(D)** MRTF-A to confirm the functional specificity of the MRTF-A antibody (Ab). Presented are blots from 1 of 3 similar experiments. **(E, F)** Western blotting of acetylated MRTF-A and its total protein, respectively. Shown are representative blots from 1 of 3 similar experiments. For ex vivo treatment of arteries with tubastatin A, rat aortas deprived of endothelium were cut into strings and cultured in Dulbecco’s modified Eagle’s medium/F12 containing 0.5% FBS. After incubation in the presence of vehicle or 10 μmol/l tubastatin A for 24 h, the artery explants were **(E)** pooled and homogenized for IP and then IB or **(F)** directly used for IB. **Dashed box** indicates shorter exposure as opposed to longer exposure of the same blot **(upper, indicated by arrow)**. **Dashed blue line** separates Input and IP on the same blot. Abbreviations as in Figures 1 and 2.

It was unanticipated that HDAC6, a primarily cytosol-residing enzyme (other HDACs are generally nuclear localized), regulates the nuclear activity of a master transcription factor (SRF). It is therefore particularly intriguing to identify the factor that could bridge the HDAC6 activity and the SRF nuclear function. MRTF-A, another master regulator of contractile gene expression, seemed to meet this role because it usually resides in the cytosol but can bind and activate SRF to initiate the transcription of SMC markers once translocated into the nucleus (20). To explore a possible regulation of MRTF-A by HDAC6, we first determined the effect of tubastatin A pretreatment on the nuclear/cytosolic ratio of MRTF-A but observed no significant changes (data not shown). However, our data showed that tubastatin A increased MRTF-A total protein (Figure 3A). We then determined acetylation levels of MRTF-A, as acetylation generally contributes to protein stabilization (7). Considering that there are numerous acetylated proteins in the cell lysate milieu, we performed immunoprecipitation to specifically pull down MRTF-A. Interestingly, our data showed that pre-treatment with tubastatin A robustly increased MRTF-A acetylation (Figure 3B). This result raised the possibility that HDAC6 may serve as an enzyme that deacetylates MRTF-A. Indeed, co-immunoprecipitation using an MRTF-A antibody specifically pulled down HDAC6 compared with IgG control (Figure 3C). The strong signal of MRTF-A pulldown relative to IgG control confirmed the specificity of the MRTF-A antibody (Figure 3D). The co-immunoprecipitation evidence for an HDAC6/MRTF-A association is consistent with a possible enzyme/substrate interaction.

To the best of our knowledge, the result that HDAC6 inhibition elevates acetylation and total protein levels of MRTF-A is a novel finding that has not been previously reported. Furthermore, to assess how HDAC6 inhibition regulates MRTF-A in the arterial wall, we performed ex vivo experiments by incubating rat aorta explants with tubastatin A. As shown in Figure 3E, tubastatin A drastically increased α-tubulin acetylation, as detected with the explant homogenates, confirming the effectiveness of this HDAC6 inhibitor. More importantly, this treatment prominently enhanced MRTF-A acetylation, similar to the in vitro result (Figure 3B). In addition, incubation of the artery explants with tubastatin A increased MRTF-A total protein and also slightly up-regulated α-SMA (Figure 3F).

### HDAC6 inhibitor (but not HDAC3 inhibitor) mitigates neointimal lesion in the restenosis model of rat artery balloon angioplasty

Although pan-HDAC inhibitors (e.g., TSA and scriptaid) have been shown to inhibit neointima, it remains unknown whether inhibition of individual HDACs affords the same benefit. A recent paper showed that systemically delivered tubastatin A reduced IH in a mouse ligation model by negating a Toll-like receptor 2–mediated phosphatidylinositol 3-kinase–Akt/p-HDAC6 pathway (21). This ligation model is heavily influenced by inflammation and more relevant to atherosclerosis. Moreover, to our knowledge, there is no report testing the effect of selective HDAC3 inhibitors on IH, although it has been reported that endothelial cell–specific HDAC3 depletion strongly enhances IH (10). To compare the effects of tubastatin A and RGFP966 on neointima, we used a rat carotid angioplasty model to induce IH and perivascular local delivery to minimize possible systemic complications. Western blotting with artery tissues showed an increase in HDAC6 in the injured arterial wall compared with uninjured control (Supplemental Figure 4), suggesting that HDAC6 could be targeted by applying tubastatin A around the injured vessel.

**Figure 4:**
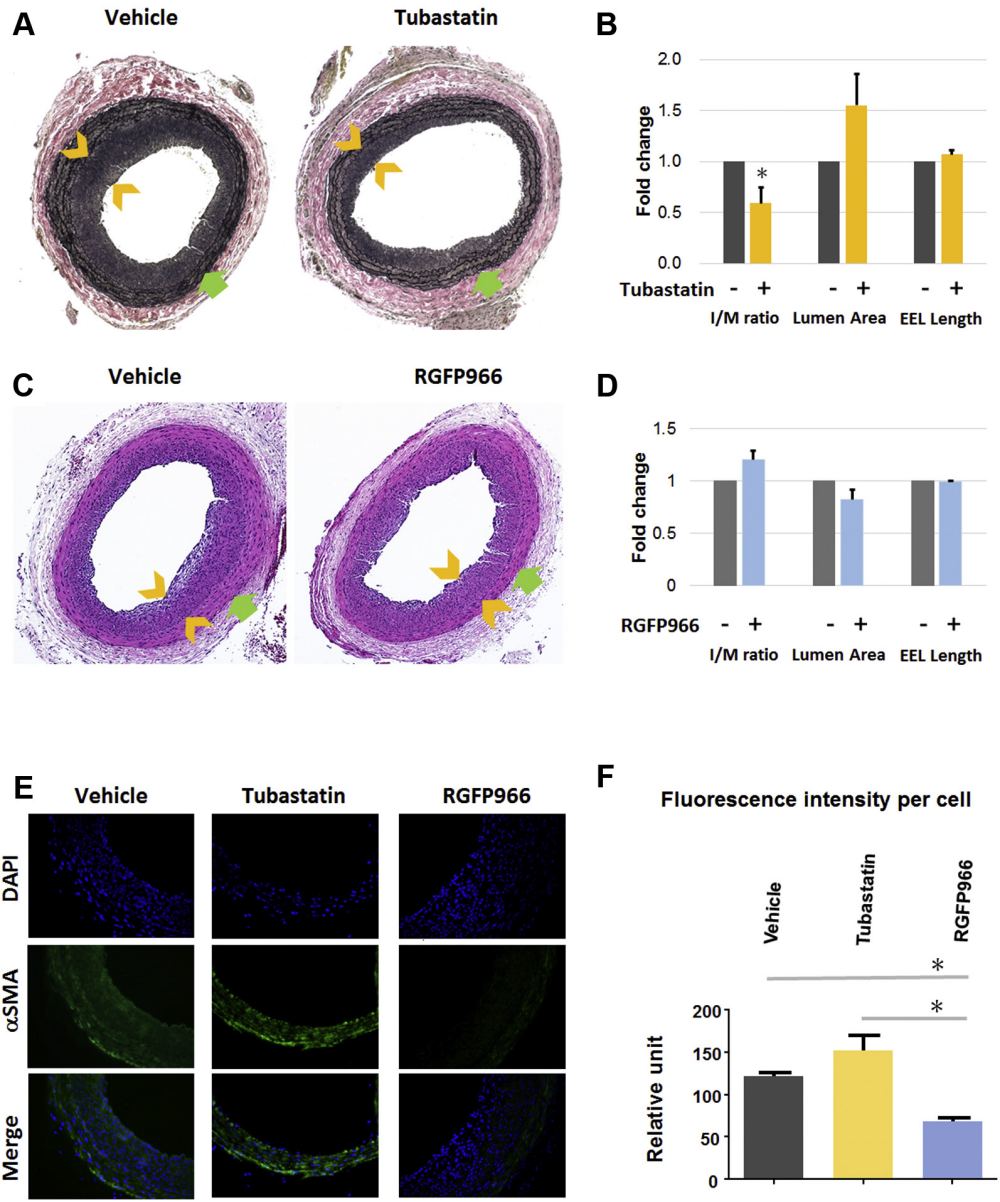
Attenuation of IH by the HDAC6 Inhibition in a Rat Restenosis Model Balloon angioplasty was performed in rat carotid arteries to induce intimal hyperplasia (IH) and restenosis (lumen narrowing), and vehicle (equal-amount dimethylsulfoxide) or tubastatin A (2 mg/rat) or RGFP (2 mg/rat) was applied in a Triblock hydrogel. Arteries were collected 14 days later for morphometric analysis. **(A and C)** Representative van Gieson– and hematoxylin and eosin–stained cross sections, respectively. Neointimal thickness is indicated between **arrowheads**; **arrow (green)** points to external elastic lamina (EEL). **(B and D)** Quantification of IH (intima/media area [I/M] ratio), lumen area, and EEL length. Data are quantified as fold changes versus vehicle control (normalized value as 1); mean ± SEM; n = 3 to 5 animals; *p < 0.05 versus control (one-sample Student’s *t*-test). **(E, F)** α-SMA immunostaining and quantification. A threshold of fluorescence intensity was set with ImageJ software to exclude the adventitia layer. Fluorescence in each image was then normalized to the total number of 4′,6′-diamidino-2-phenylindole (DAPI)-stained nuclei in the medial and neointimal layers, which was manually counted. Data presentation: mean ± SEM; n = 3 to 5 animals; *p < 0.05 (1-way analysis of variance). Abbreviations as in Figures 1 and 2.

We found that 2 weeks after balloon angioplasty, treatment with tubastatin A decreased IH (measured as the intima/media area ratio) by 40% and increased lumen size by up to 60% compared with vehicle control (Figures 4A and 4B). There was no difference in the overall vessel size measured as EEL length. These data indicate that perivascular application of tubastatin A effectively mitigated restenosis without causing constrictive remodeling (reduced EEL length). In contrast, RGFP966 slightly increased IH and decreased lumen size, although the changes were not statistically significant (Figures 4C and 4D). We also tried a different hydrogel (Pluronic gel) to deliver RGFP966, and Apicindin, another commonly used selective HDAC3 inhibitor, but neither experiment resulted in neointimal inhibition (data not shown). Therefore, the results indicate that HDAC6 inhibitor, but not HDAC3 inhibitor, effectively mitigates IH.

Finally, the effect of HDAC inhibition on α-SMA in vivo was determined by immunostaining artery cross sections. Consistent with the in vitro result (Figure 1A), treatment with tubastatin A increased α-SMA in the arterial wall (including media and neointima) by ∼30% relative to vehicle control, albeit without statistical significance (Figures 4E and 4F). On the contrary, treatment with RGFP966 significantly reduced α-SMA by ∼50%.

## Discussion

In the present study, we made an unanticipated finding that inhibition of HDAC6, which is best known as cytosolic localized, enhances SRF nuclear activity and preserves SMC contractile protein levels. Mechanistically, another novel finding was that HDAC6 inhibition increases acetylation and total protein levels of MRTF-A, a powerful co-factor that resides in the cytosol but is able to translocate to the nucleus to activate the SRF transcriptional activity in contractile gene expression. To the best of our knowledge, these HDAC6 functions have not been previously reported in any cell type or tissue. Inasmuch as HDAC6 inhibition also attenuates SMC proliferation/migration and inflammation, controlling HDAC6 activity may open a new method to mitigate neointimal growth by abrogating “full-spectrum” SMC pathogenic phenotypes.

The somewhat surprising findings presented herein resulted from our experiments to initially compare HDAC6 and HDAC3 functions using isoform-selective inhibitors. Studies have explored the role of HDACs in SMC phenotypic transformation, including those with TSA 11, 12, 13, 14, 16, a pan inhibitor that blocks the activities of class I and class II (but not class III) HDACs. In these studies, SMC proliferation and migration were determined, but SMC inflammation and dedifferentiation were generally left unexplored. Given our finding that inhibiting HDAC6 (class IIb) up-regulates SMC markers, it is seemingly contradictory that TSA reduced these proteins in a previous study (16). However, our data also showed that inhibiting HDAC3, a class I member, reduced α-SMA, consistent with the previous report. As the outcome of using a pan-HDAC inhibitor represents the sum of the effects mediated by all the targeted HDACs, the gross effect of a pan inhibitor may vary depending on multiple factors (e.g., concentration or treatment duration of the inhibitor). We hence speculate that in this previous study using TSA (16), the effect of HDAC3 inhibition by TSA may have overridden that of HDAC6 inhibition. Therefore, discrepancies between the results from using selective and pan inhibitors strongly advocate the importance of studying HDACs individually to dissect their differential regulations.

Our finding is novel, especially considering that HDAC6 is poorly characterized for its functions in vascular pathobiology. In particular, the impact of selectively inhibiting HDAC6 on SMC marker proteins has not been previously reported. Earlier studies tackled the molecular mechanisms of SMC phenotype switching (or transformation) involving HDACs 2, 4 and 5 (15), yet with HDACs 3 and 6 unaddressed. These studies found that under PDGF-BB stimulation, KLF4 recruits HDAC2, 4, or 5 to the SMC marker genes blocking their transcription via histone (H4) deacetylation. It is interesting to note that although HDACs 2 and 3 both belong to class I, they seem to regulate SMC marker genes in opposite directions. In this regard, down-regulation of SMC markers by HDAC3 inhibition (Figure 1) is an intriguing novel observation as well. The differential mechanisms deserve further investigation. Recently, the HDAC6 inhibitor tubastatin A was shown to inhibit neointima in a mouse carotid ligation model, but it was not addressed as to whether SMC contractile genes were regulated (21). In another recent study, down-regulation of HDAC6 (protein and activity) by NogoB silencing correlated with reduced α-SMA and reduced migration but enhanced proliferation of rat primary aortic SMCs (14). Apparently, these results differ from our observations here made by directly inhibiting HDAC6 with tubastatin A or siRNA. It is unclear whether the reported SMC phenotype changes resulted indirectly from non-HDAC6 pathways downstream of NogoB or directly from HDAC6 nullification.

Although tubastatin A binds HDAC6 with a >1,000-fold selectivity over all other HDACs, there is an exception: its selectivity for HDAC6 over HDAC8 is only 57-fold. Thus, a question arises as to whether the outcomes from tubastatin A pre-treatment in our study were mediated by HDAC8. This scenario is unlikely in light of the following facts. First, HDAC6 and HDAC8 belong to different classes (IIb and I, respectively) with distinct domain structures. Second, our data show that HDAC6 silencing with siRNA largely recapitulated the tubastatin A effect in activating SRF. Third, several reports have shown that HDAC8 associates with actin filaments, promoting their assembly and α-SMA production (22). On the basis of this conclusion, if tubastatin A had inhibited HDAC8 instead of HDAC6 in our study, it would have reduced α-SMA production; our observation was the opposite, however.

HDAC6 is unique among all HDACs not only because it is primarily located in the cytosol but also for its well-established function in tubulin deacetylation and hence microtubule destabilization (4). A new study using RPE1 cells showed that the production of α-SMA mediated by the MRTF-A/SRF axis positively regulates microtubule stability (23). However, whether microtubule stability reciprocally influences α-SMA production remains an open question. Our result is consistent with the literature in that pretreatment of SMCs with tubastatin A dose-dependently enhanced tubulin acetylation. However, this effect on tubulin cannot explain the increased α-SMA after HDAC6 inhibition (Figures 1 and 4) because a commonly used microtubule destabilizer, colchicine, reportedly did not reduce, but increased, α-SMA (24). We thus explored alternative pathways and found that inhibiting HDAC6 elevated MRTF-A acetylation and protein levels.

To the best of our knowledge, there has been no literature evidence pointing to a role of HDAC6 in MRTF-A deacetylation, although a number of substrates of the HDAC6 deacetylase have been proposed, including tubulin, HSP90, cortactin, and survivin 3, 4. Unlike myocardin, which is a nuclear protein, MRTF-A is sequestered in the cytosol by G-actin and is released when G-actin polymerizes into F-actin filaments. Free MRTF-A is able to translocate into the nucleus to bind SRF, thereby activating the transcription of SMC marker genes (15). Our data show that although total MRTF-A protein was increased after HDAC6 inhibition, the nuclear/cytosolic ratio of MRTF-A distribution was not significantly altered (data not shown). We thus infer that due to inhibited HDAC6 enzymatic activity, more MRTF-A proteins stay acetylated and stabilized. This outcome may have proportionally increased nuclear MRTF-A, which together with SRF coactivates the transcription of SMC marker genes. Importantly, we indeed observed that HDAC6 inhibition augmented the luciferase signal, indicating enhanced SRF-directed transcription. Moreover, because both HDAC6 and MRTF-A are primarily cytosol localized, it is not unreasonable to speculate that HDAC6 may catalyze deacetylation on the MRTF-A protein regulating its stability. It is known that deacetylated proteins are generally susceptible to ubiquitination and proteasomal degradation (7). Intriguingly, our data indicated specific pulldown of HDAC6 via co-immunoprecipitation by using an MRTF-A antibody, implicating an HDAC6/MRTF-A interaction. In view of the reported role of HDACs (2, 4, and 5) in suppressing SMC marker genes via chromatin remodeling (15), the up-regulation of α-SMA and SMHC observed here involving elevated MRTF-A acetylation likely represents a unique and novel HDAC6-mediated mechanism. Thus, more detailed future studies are important for elucidating this mechanism in order to develop HDAC6-targeted interventions.

Neointima constitutes flow-obstructing lesions on the vessel wall and is hence the principal etiology of the major vascular diseases such as atherosclerosis and restenosis. SMC pathogenic transformation, manifested by acquired phenotypes including proliferation/migration, inflammation, and dedifferentiation, has been recognized as the key causative event in neointimal development 2, 17. In this regard, HDAC6 inhibition affords a favorable strategy for effective mitigation of neointima, as our results indicate that the HDAC6 inhibitor abates all the aforementioned pathogenic SMC phenotypes. In contrast, HDAC3 inhibitor did not preserve but rather disrupted a normal differentiated state of SMCs. This “defect” may partially explain the lack of inhibitory effect of RGFP966 on neointima. Moreover, in addition to the normal SMC state, the well-being of endothelial cells is also important in attenuating neointimal development 10, 17. Aside from the benefits of HDAC6 inhibition for blocking SMC phenotypic transformation, recent literature indicates endothelial cell protection by HDAC6 inhibition (21). On the contrary, compelling evidence indicates that endothelial cell–specific HDAC3 depletion substantially enhances IH in an atherosclerosis mouse model (10). Taken together, our in vitro and in vivo results and the literature evidence suggest that HDAC6 inhibition, but not HDAC3 inhibition, could give rise to a viable paradigm for averting neointimal pathogenesis. A potential caveat of α-SMA up-regulation via HDAC6 inhibition would be excessive α-SMA accumulation. However, treatment with tubastatin A only reinstated α-SMA that was diminished by PDGF-BB in cultured cells (Figure 1A) and only slightly increased α-SMA either ex vivo in artery explants (Figure 3) or in vivo in the balloon-injured arterial wall (Figure 4). Nonetheless, the HDAC6-targeting paradigm for mitigating aberrant vascular cell/wall remodeling warrants more thorough future evaluations.

### Study limitations

While we found that HDAC6 inhibition increases acetylated MRTF-A, it will take more detailed biochemical work to prove or disprove MRTF-A as a novel substrate of HDAC6. In the preclinical tests, the observed neointimal mitigation by Tubastatin A may not be optimal, considering that the outcomes of in vivo experiments are influenced by many factors, such as drug solubility, dosing, and variability of drug/carrier interactions.

## Conclusions

Although gross effects of pan-HDAC inhibitors on vascular cell pathobiology and neointima have been investigated, functional mechanisms of individual HDACs, especially the class IIb protein HDAC6, have been poorly differentiated. Inspired by our initial finding that inactivating HDAC6 maintains SMC marker protein levels under pathogenic stimulation, we have further tracked down a mechanism whereby HDAC6 inhibition enhances MRTF-A protein acetylation and abundance and also SRF transcriptional activity downstream of MRTF-A. These new insights, along with the benefits afforded by HDAC6 blockage to inhibit SMC transformation and neointima, advocate an HDAC6-targeting therapeutic paradigm. Validation of this paradigm in treating vascular diseases requires more in-depth future research.

Perspectives **COMPENTENCY IN MEDICAL KNOWLEDGE:** De-differentiated SMCs lose contractile function and perpetrate pathologies. It is thus important to restrain this phenotypic instability for effective treatment of vascular diseases such as atherosclerosis and restenosis. We found that tuning down the activity of HDAC6, a unique acetyl-erasing enzyme, blocks SMC dedifferentiation. This study implicates potential interventions to preserve a normal, differentiated SMC state for abrogation of vascular pathogenesis.

**TRANSLATIONAL OUTLOOK 1:** Although tubastatin A exhibited antirestenotic effectiveness here in small animals, more knowledge on its safety profile is needed to evaluate its potential for clinical use.

**TRANSLATIONAL OUTLOOK 2:** Future research is warranted to carefully examine the effects of HDAC6 inhibition on the homeostasis of not only SMCs but also endothelial cells and adventitial fibroblasts.
