# Supplementary material for: HDAC6 Regulates the MRTF-A/SRF Axis and Vascular Smooth Muscle Cell Plasticity
Source: JACC Basic Transl Sci. 2018 Dec 31;3(6):782–95. doi: 10.1016/j.jacbts.2018.08.010 (PMC6314972; doi:10.1016/j.jacbts.2018.08.010)
Supplement: Supplemental Figures 1–4 [file mmc1.pdf]

Supplemental Figure 1

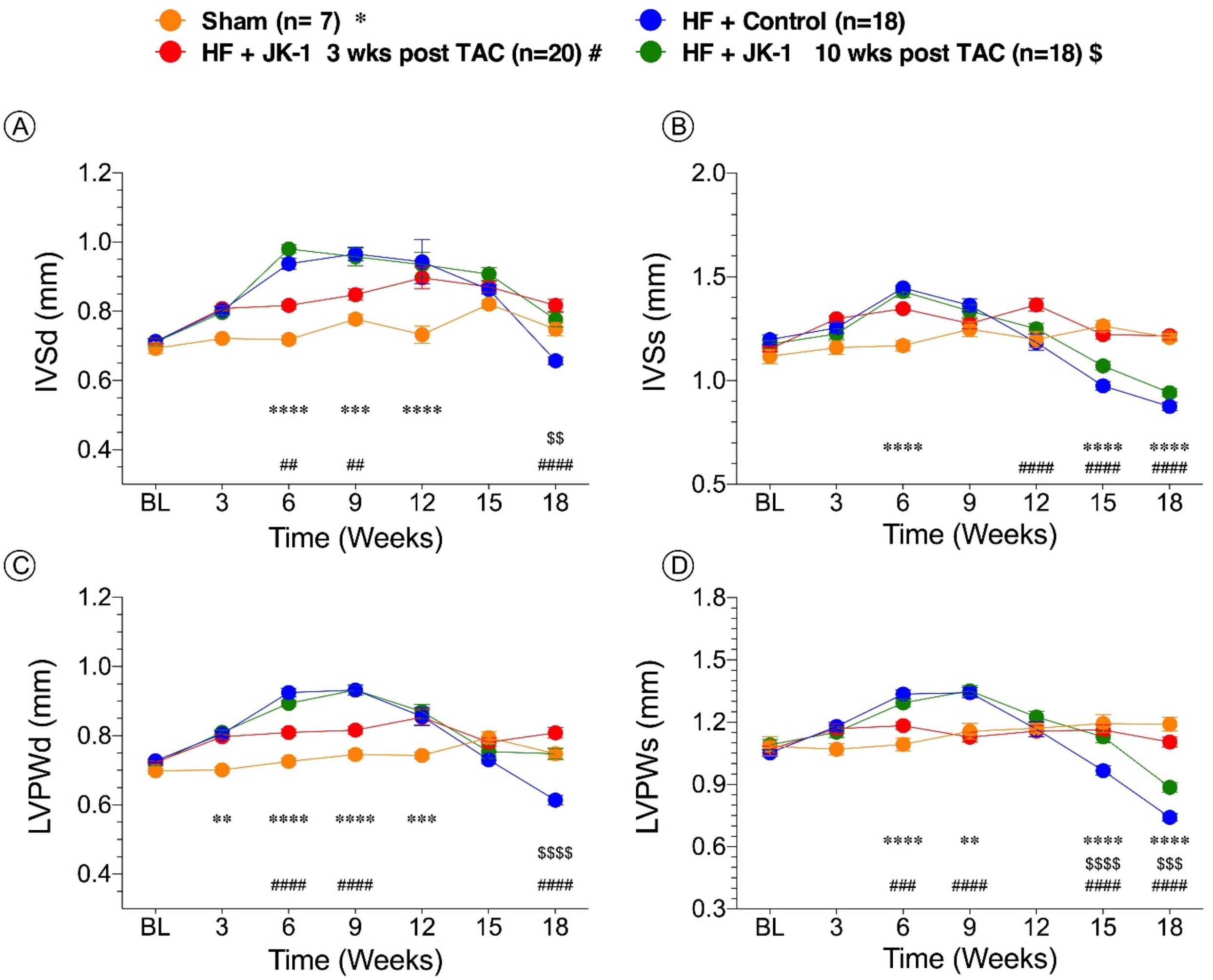

Supplemental Figure 1. **Interventricular Septal Wall and LV Posterior Wall Thickness**
Interventricular wall thickness at diastole (***A***) and at systole **(*B*)**; left ventricular posterior wall at diastole (***C***) and at systole (***D***) in mice from Sham, HF + Control, HF + JK-1 3 weeks post TAC, and HF + JK-1 10 weeks post TAC groups. **: p < 0.01 between Sham vs. HF + Control; ***: p < 0.001 between Sham vs. HF + Control; ****: p < 0.0001 between Sham vs. HF + Control; #: p < 0.01 between HF + JK-1 3 weeks post TAC vs. HF + Control; ###: p < 0.001 between HF + JK-1 3 weeks post TAC vs. HF + Control; ####: p < 0.0001 between HF + JK-1 3 weeks post TAC vs. HF + Control; $$: p < 0.01 between HF + JK-1 10weeks post TAC; $$$: p < 0.001 between HF + JK-1 10weeks post TAC; $$$$: p < 0.0001 between HF + JK-1 10weeks post TAC. All data in ***Supplemental Figure 1*** were analyzed with two-way-ANOVA.

Supplemental Figure 2

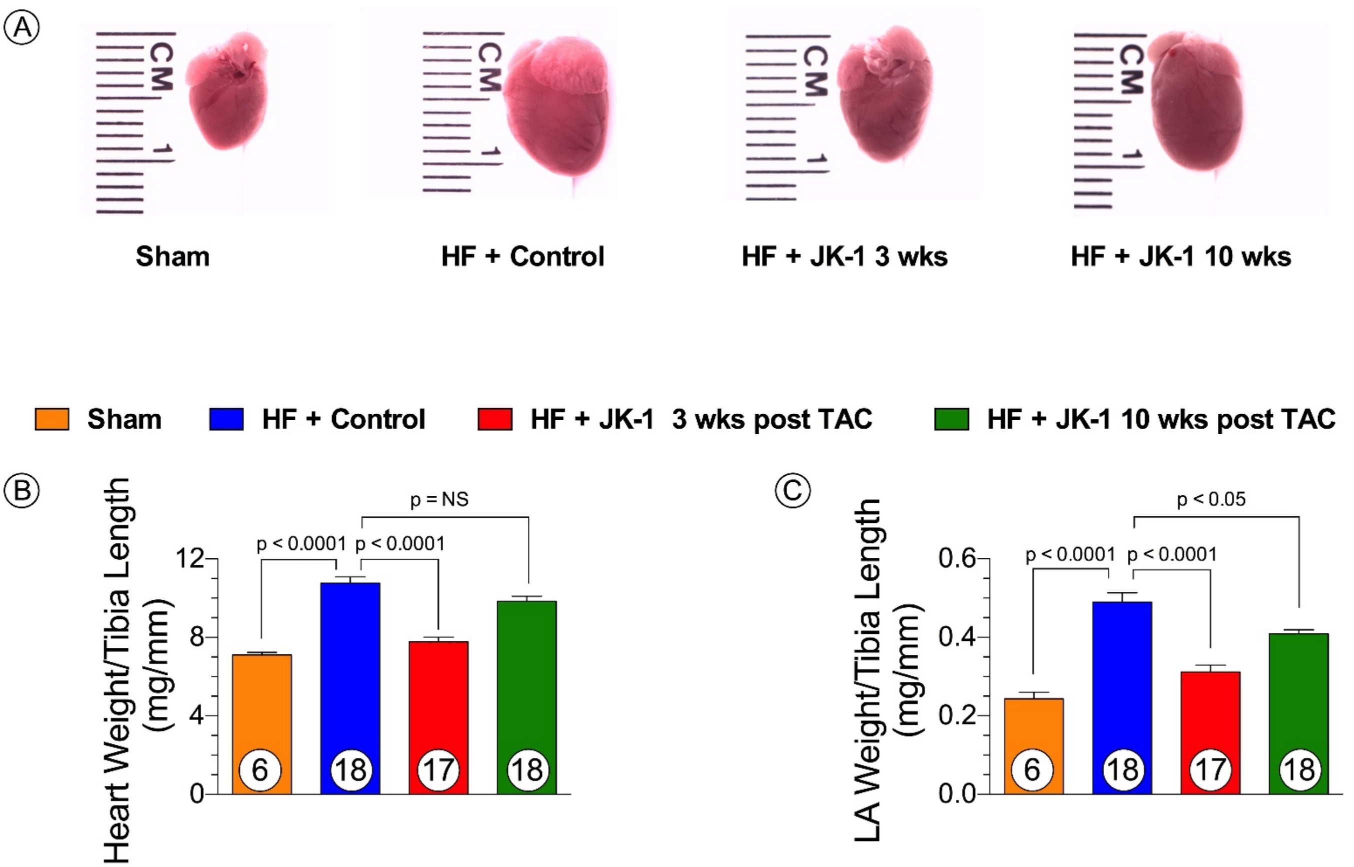

Supplemental Figure 2. **Pathological Cardiac Remodeling, Whole Heart Weight to Tibia Length Ratio, and Left Atrial Weight to Tibia Length Ratio**

(***A***) Representative images of the whole heart, (***B***) whole heart weight to tibia length ratio, and (***C***) left atrial weight to tibia length ratio in mice from Sham, HF + Control, HF + JK-1 3 weeks post TAC, and HF + JK-1 10 weeks post TAC groups at 18 weeks post-TAC. Circles inside bars denote the sample size.

Supplemental Figure 3

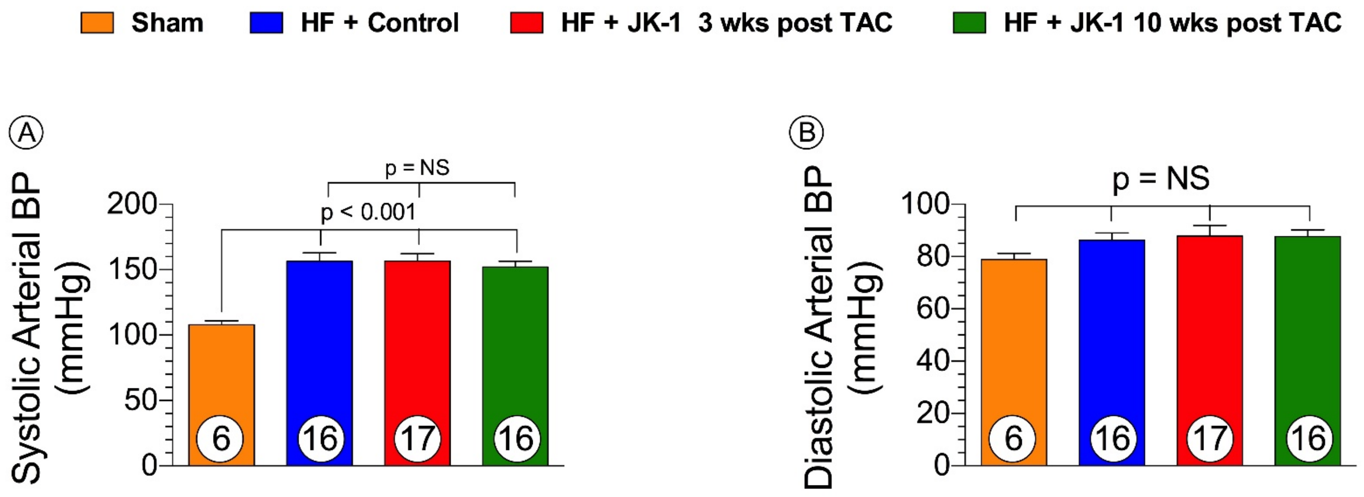

Supplemental Figure 3. **Systolic and Diastolic Arterial Blood Pressure**

(A) Systolic and (***B***) diastolic arterial blood pressure from Sham, HF + Control, HF + JK-1 3 weeks post TAC, and HF + JK-1 10 weeks post TAC groups at 18 weeks post-TAC. Circles inside bars denote the sample size.

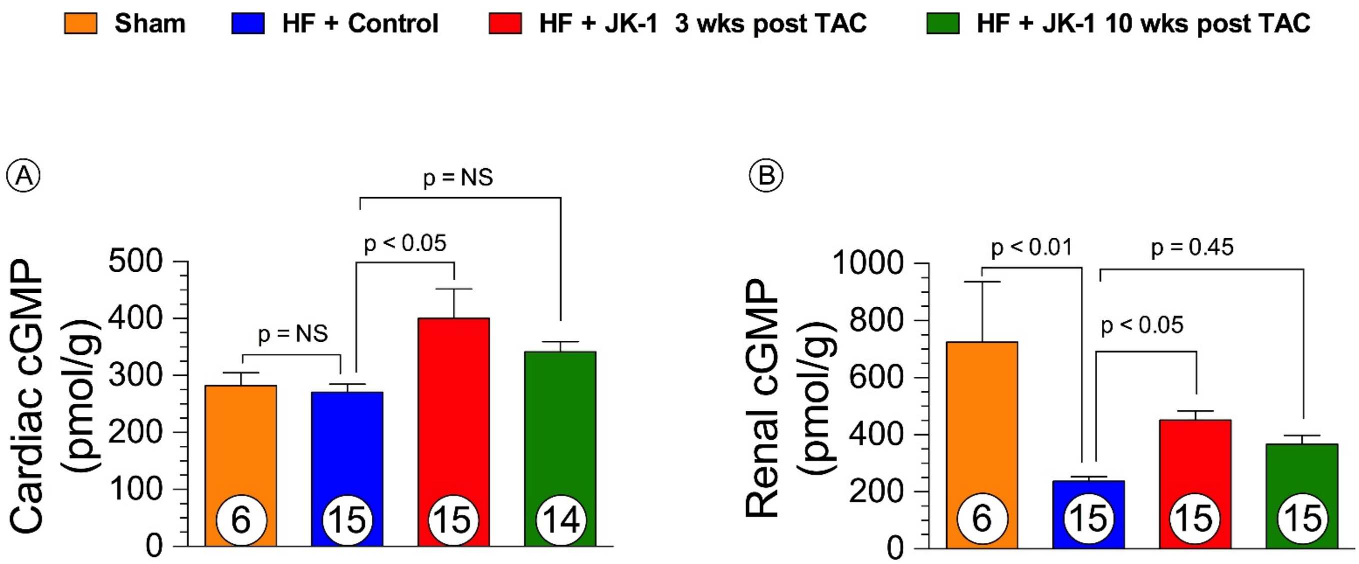

Supplemental Figure 4. **Cardiac and Renal cGMP levels**
Levels of cGMP in (***A***) cardiac and (***B***) renal tissues from Sham, HF + Control, HF + JK-1 3 weeks post TAC, and HF + JK-1 10 weeks post TAC groups at 18 weeks post-TAC. Circles inside bars denote the sample size.
